# Short term effects of contralateral tendon vibration on motor unit discharge rate variability and force steadiness in people with Parkinson’s disease

**DOI:** 10.3389/fnagi.2024.1301012

**Published:** 2024-03-04

**Authors:** Changki Kim, Daryl J. Wile, Sarah N. Kraeutner, Kaylee A. Larocque, Jennifer M. Jakobi

**Affiliations:** ^1^Faculty of Health and Social Development, University of British Columbia Okanagan, Kelowna, BC, Canada; ^2^Healthy Exercise and Aging Laboratory, Aging in Place Research Cluster, University of British Columbia Okanagan, Kelowna, BC, Canada; ^3^Centre for Chronic Disease Prevention and Management, University of British Columbia Okanagan, Kelowna, BC, Canada; ^4^Department of Psychology, University of British Columbia Okanagan, Kelowna, BC, Canada

**Keywords:** motor neurons, electromyography, tremor, muscle rigidity, recruitment

## Abstract

**Background:**

Vibration of one limb affects motor performance of the contralateral limb, and this may have clinical implications for people with lateralized motor impairments through vibration-induced increase in cortical activation, descending neural drive, or spinal excitability.

**Objective:**

The objective of this study was to evaluate the effects of acute biceps brachii tendon vibration on force steadiness and motor unit activity in the contralateral limb of persons with Parkinson’s disease.

**Methods:**

Ten participants with mild to moderate Parkinson’s disease severity performed a ramp, hold and de-ramp isometric elbow flexion at 5% of maximum voluntary contraction with the more-affected arm while vibration was applied to the distal biceps brachii tendon on the contralateral, less-affected arm. Using intramuscular fine wire electrodes, 33 MUs in the biceps brachii were recorded across three conditions (baseline, vibration, and post-vibration). Motor unit recruitment & derecruitment thresholds, discharge rates & variability, and elbow flexion force steadiness were compared between conditions with and without vibration.

**Results:**

Coefficient of variation of force and discharge rate variability decreased 37 and 17%, respectively in post-vibration compared with baseline and vibration conditions. Although the motor unit discharge rates did not differ between conditions the total number of motor units active at rest after de-ramp were fewer in the post-vibration condition.

**Conclusion:**

Contralateral tendon vibration reduces MU discharge rate variability and enhances force control on the more affected side in persons with Parkinson’s disease.

## 1 Introduction

Acute mechanical vibration is an effective way of altering afferent feedback, and corticospinal excitability (Lapole et al., 2015b; Li et al., 2019) and offers the potential to ameliorate impaired sensory information processing and deficits in upper-limb motor performance (Abbruzzese and Berardelli, 2003; Aman et al., 2015). When vibration (5–180 s) is applied acutely in healthy adults to the ipsilateral (Martin and Park, 1997; Grande and Cafarelli, 2003; Harwood et al., 2014; Lapole et al., 2015b) or contralateral (Lapole et al., 2015b; Li et al., 2019) side isometric force steadiness improves during (Germer et al., 2020) and following (Muceli et al., 2011; Harwood et al., 2014) the application of the mechanical stimulus. Force steadiness is an explanatory variable of clinical hand-dexterity screening tests and functional movement, thereby offering an elemental understanding of the neurophysiology of complex dynamic movements from measures of isometric single joint tasks (Almuklass et al., 1985; Marmon et al., 2011; Davis et al., 2020). Vibration-induced enhancement in isometric force steadiness is ascribed to adaptations in spinal output through alteration in 1A afferents uncoupling oscillatory input to coactive muscles (Muceli et al., 2011; Harwood et al., 2014; Lapole et al., 2015b, 2023), and reductions in variance of the common synaptic input to the motor neuron pool (Lapole et al., 2015a; Germer et al., 2020).

In persons with Parkinson’s disease (PD) there is an increase in motor unit (MU) doublets/triplets and synchrony (Dietz et al., 1974), discharge rate variability in the finger extensors (Freund et al., 1973; Dietz et al., 1974; Dengler et al., 1986), elbow flexors and extensors (Wilson et al., 2020), and force steadiness is reduced compared to healthy controls (Skinner et al., 2019; Smart et al., 2020). Even in the early stages of PD hand and upper limb dysfunction are common symptoms (Corcos et al., 1996; Kandaswamy et al., 2018; Dan et al., 2019). Although PD associated movement disorders and tremors are typically ascribed to supraspinal dopaminergic origins recent reports suggest that spinal mechanisms play a role in motor disruptions, and that afferent input from muscle spindles contribute (Anastasopoulos, 2020). Reminiscent of the vibration induced motor performance benefit in healthy adults (Muceli et al., 2011), short duration acute vibration (80 Hz) also improves hand function in persons with PD (Macerollo et al., 2018).

Since the pioneering work of Goodwin et al. (1972) we know tendon vibration activates the muscle spindle primary endings and induces instantaneous afferent activity, that recently was suggested to culminate in serotonergic neuromodulation of intrinsic motoneuron excitability (Lapole et al., 2023). This vibration prompted adaptation is most often studied in the muscle associated to the mechanical perturbation on the ipsilateral side yet, the neuromodulators bathe the entire spinal milieu. The contralateral side to vibration is often overlooked. The commissural system is an essential component of the spinal motor circuits (Maxwell and Soteropoulos, 2020; Kinany et al., 2023).

With improved understanding of nondopaminergic dysfunction in PD, including serotonergic influences on motor and nonmotor symptoms (Qamhawi et al., 2015), we wonder if there is particular effect of sensory afferent input on spinal motor unity activity in people with PD, which might manifest at a segmental (e.g., bilateral) spinal level. The objective of this study was to evaluate the effects of acute contralateral biceps brachii tendon vibration on force steadiness and MU activity in persons with PD. Herein, we used intramuscular fine wire electrodes to follow the MUs between vibration and non-vibration conditions in an isometric force tracking task. We hypothesized that tendon vibration would improve force steadiness and decrease MU discharge rate variability on the contralateral side in persons with PD.

## 2 Methods

### 2.1 Participants

Six males (66.17 ± 6.85 yrs) and four females (69.75 ± 1.89 yrs) with mild to moderate PD severity (stages I-III; Hoehn and Yahr, 1967; Table 1) participated in this study within one-hour of taking dopaminergic medication. Individuals with severe motor disability (Hoehn & Yahr IV-V), severe cognitive impairment, cardiorespiratory comorbidities or neurological disorders other than PD were excluded. The University Research Ethics Board (H11-01931) approved the experimental procedures and participants provided written informed consent prior to participation.

**Table 1:** Participant characteristics.

	Males (*n* = 6)	Females (*n* = 4)	Overall (*n* = 10)
Age (yrs)	66.17 ± 6.85	69.75 ± 1.89	67.60 ± 5.54
Height (cm)	173.99 ± 9.06	161.12 ± 3.18	168.84 ± 9.65
Mass (kg)	79.54 ± 9.20	58.63 ± 8.68	71.17 ± 13.74
Hoehn Yahr Rating	2.33 ± 0.52	1.50 ± 0.58	2.00 ± 0.67
PD Diagnosis Duration (yrs)	2.63 ± 3.17	3.25 ± 1.50	2.88 ± 2.54

Values are means ± SD. Yrs, years; cm, centimeter; kg, kilogram.

### 2.2 Experimental set-up

**Figure 1:**
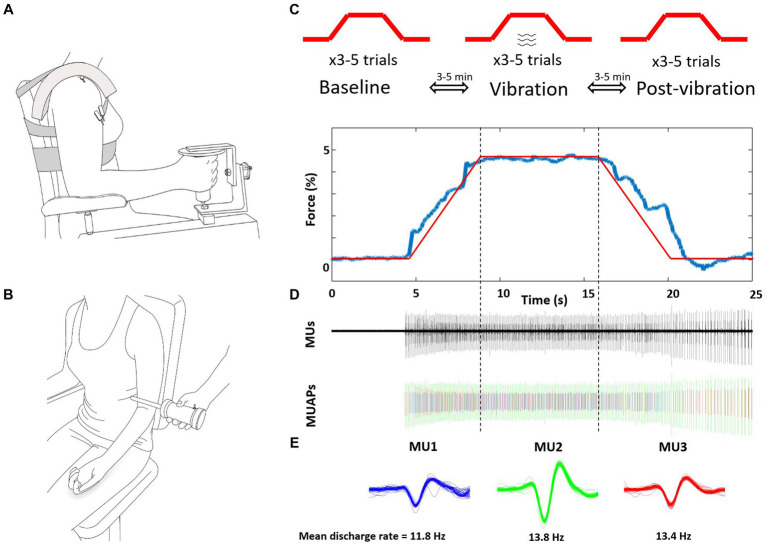
Schematic representation of the experimental setup and protocol. **(A)** Intramuscular fine wires were inserted into the biceps brachii of the more-affected arm. **(B)** Vibration was applied to the biceps brachii tendon during the ramp, sustained contraction and de-ramp of the less-affected contralateral arm. **(C)** The 5% isometric ramp contraction tasks were performed 3x consecutively for three conditions of baseline (without vibration) vibration, and post-vibration (without vibration) with 3–5 min of no vibration rest between contractions. **(D)** Representative force, detected MUs, and separated MU action potentials of one post-vibration trial. **(E)** Three MUs (different colors) were extracted from this trial, and were tracked across the three conditions. Mean discharge rates were calculated between vertical dotted lines, from 7.5 s to 17.5 s and the middle 5 s reported to account for transition to and off the plateau. MUs, motor units; MUAPs, discriminated.

Elbow flexion force and biceps brachii muscle activity of the self-identified more-affected side were recorded in the neutral forearm position (Figure 1A) while vibration was applied to the distal biceps brachii tendon of the contralateral less-affected arm (Figure 1B). Participants were seated in a custom-built dynamometer chair, with the hand of the more affected arm grasping the force manipulandum (Model: MLP150, Transducer Techniques, Temecula, CA, USA). The elbow was positioned at 90°, shoulder abducted 20° and 0° forward flexion.

#### 2.2.1 Force and intramuscular electromyography data acquisition

Isometric elbow flexion force was acquired at 1000 Hz, and analog to digital converted (Power 1401; Cambridge Electronic Design (CED), Cambridge England), and then stored for offline analysis. Elbow flexor contractile properties of the more-affected side were measured by applying percutaneous electrical stimulation through carbon rubber stimulation electrodes (4 × 4.5 cm) placed over the muscle belly. To elicit twitches at rest, 50 μs single pulses (1 Hz) were applied (DS7AH: Digitimer. LTD. Welwyn Garden City, UK).

Custom-made fine wire electrodes (125 μm in diameter; 3–5 cm in length) were inserted into the long head (LH) and the short head (SH) of the biceps brachii on the more-affected side. At the tips of each of these fine wires a hook was placed to increase stability of placement. At the ends of the wires not inserted into the muscle a finite area of insulation was removed, and the wire was abraded with sandpaper. The common ground electrode was placed over the carpometacarpal joint of the wrist. Intramuscular fine wires were amplified (100–1,000×, Neurolog, Welwyn Garden City, UK), and filtered (10Hz5kHz; Neurolog NL824 and NL135, respectively, Welwyn Garden City, UK) at an acquisition rate of 12,000 Hz (1401 Plus; CED, Cambridge, England).

#### 2.2.2 Tendon vibration

Vibration was applied across the distal biceps brachii tendon at mid-point of the less-affected arm with a custom-made vibration apparatus (100 Hz Frequency; ~3.5 mm amplitude; Don Clarke, University of Windsor, ON, Canada) (Figure 1B). To landmark the vibration position, participants performed brief elbow flexion and the center of the distal biceps brachii tendon was palpated and marked.

### 2.3 Experimental procedure

#### 2.3.1 Maximal voluntary contraction (MVC) and twitch interpolation

Current intensity was increased until twitch force plateaued, and then increased an additional 10%. After 3–5 practices, isometric elbow flexion MVC was measured and active verbal encouragement given. Participants were instructed to flex the forearm upward as fast and as hard as possible, and maintain the contraction for 3–5 s. Twitch stimuli were delivered prior to (resting twitch), during (superimposed twitch) and following the MVC (potentiated twitch) to determine voluntary activation (VA) with the twitch interpolation technique (Merton, 1954; Allen et al., 1995; Jakobi and Rice, 2002). Each contraction was separated by 2–3 min of rest, and the MVC value with the highest VA score was selected to establish submaximal target forces. The MVC was re-confirmed at the end of the experimental session.

#### 2.3.2 Submaximal isometric contractions

Participants practiced the task prior to insertion of the indwelling electrodes, and then performed 3–6 submaximal contractions consecutively to track identified MUs between each of the three submaximal conditions: baseline (no vibration), vibration, post-vibration (no vibration). The target force of 5% MVC was selected because low force levels exhibit a low level of force steadiness and MUs are readily tracked between separate trials. Between trials ~3 min of rest was provided and between conditions ~5 min of rest occurred (Figure 1C). The submaximal task consisted of a 5 s ramp (increasing force), a 10 s hold (5% plateau), and a 5 s de-ramp (decreasing force) (Figure 1D). During the vibration condition, the tendon was vibrated for the entire duration by activating the vibrator before the ramp and after the de-ramp. Consistent visual feedback of force output was provided with a 20.5-inch monitor screen located 1 m in front of participant and at eye level.

### 2.4 Data analysis

#### 2.4.1 Twitch contractile properties

Twitch contractile properties were analyzed off-line (Spike 2 version 7.0, CED, Cambridge, England). VA was obtained by taking the ratio of superimposed twitch (Tw_s_) during MVC to the potentiated twitch (Tw_p_) measured after MVC and expressing it as a percentage: [(1– Tw_s_/Tw_p_) × 100 = % activation]. Post-Activation Potentiation (PAP) was determined by the difference between Tw_p_ and resting twitch (Tw_r_): [(Tw_p_–Tw_r_)/Tw_r_ × 100 = % change]. Twitch contractile properties were calculated from Tw_r_ for: (1) peak tension (PT): maximal amplitude of the twitch (N); (2) time to peak tension (TPT): time duration from the rise in twitch force to peak tension (ms); (3) half relaxation time (HRT): time duration from peak tension to half the amplitude of decreasing force (ms); and (4) contraction duration (CD): time duration of TPT and HRT (ms).

#### 2.4.2 Force steadiness (CV of force)

Force data was analyzed offline using custom-written programs in MATLAB^®^ (Math Works^™^ Inc., Natick, Massachusetts, USA). The force signal was low-pass filtered at 10 Hz with a second order Butterworth filter, and mean force and force steadiness analyzed for the plateau phase with small areas of exclusion at the onset and offset of the plateau (~0.5–1 s) excluded by visual inspection to ensure force was at the 5% target. Force steadiness was quantified as the coefficient of variation (CV of force = 100 * [SD of force / mean force]) of the force signal, and the mean value for the trials in which MUs were tracked are reported.

#### 2.4.3 Motor units

Motor units were analyzed using Spike 2 version 7.0 (CED, Cambridge, UK) over the identical time period of the force steadiness analysis (~7 s). Intramuscular recordings were visually inspected and were identified using a template-matching algorithm by waveform shape, and discharge behavior by comparing action potentials with respect to temporal and spatial characteristics (Figure 1E). MU trains that had less than five consecutive MU action potentials, or had inter-spike intervals less than 10 ms or greater than 150 ms were excluded (Barry et al., 2007; Dalton et al., 2010). MUs were matched and tracked between the three conditions (baseline, vibration, post-vibration). For each MU train, the average MUDR (Hz) and CoV_ISI_ (CV of discharge rates) during the plateau for the three conditions were determined (baseline, vibration, post-vibration) with monitoring of the MUs for approximately 1–2 s after the contraction. Recruitment thresholds (%MVC) were calculated as the level of force at which a MU train discharged its first action potential, and derecruitment thresholds (%MVC) as the level of force at which a MU ceased discharging.

### 2.5 Statistical analysis

Descriptive statistics were calculated initially for the males and females with independent t-tests for the contractile properties (PT, TPT, HRT, CD, and PAP), VA, and MVC. The MVC was also verified after the experimental protocol with a paired *t*-test. To address the primary research question of the influence of contralateral vibration the dependent variables of: (1) Recruitment and derecruitment thresholds, (2) CV of force, (3) MUDR, and (4) CoV_ISI_ were compared between the three conditions (baseline, vibration, post-vibration). Shapiro-Wilks normality test confirmed that at least one condition in all the dependent variables were not normally distributed. Thus, to compare the effect of vibration non-parametric Friedman’s tests were conducted. Wilcoxon signed-rank test was applied when significant main effects were found. Given that multi-level modeling may be more suitable for the dataset and more accurately accounting for random error, we additionally replicated our statistical analyses by conducting separate linear mixed effects models (one per CV, MUDR, and MUDRV). Such analyses resulted in the same effects observed (and thus subsequent interpretations), and are included in Supplementary materials. Spearman’s rank correlation was used to determine the association between the recruitment threshold and derecruitment threshold in the three conditions. All statistical analyses were performed with IBM SPSS Statistics (IBM Corp. Version 27.0. Armonk, NY: IBM Corp. SPSS, Inc., Chicago, IL). The alpha level for all statistical tests was 0.05 and data are reported as mean ± SD within the text and figures.

## 3 Results

### 3.1 Contractile properties

Participant characteristics of twitch contractile properties, post-activation potentiation and voluntary activation for the males and females with PD ON medication are described in Table 2. The VA scores for all participants were high (98.2 ± 1.3%), and PT (*t*_8_ = 2.98, *p* = 0.02) and MVC (*t*_8_ = 3.85, *p* = 0.005) were higher in males compared with females. The MVC did not differ from beginning to end of the experiment (before:160.93 ± 57.46 N, after:153.02 ± 53.77 N, *t*_9_ = 2.16, *p* = 0.06).

**Table 2:** Contractile properties.

	Males (*n* = 6)	Females (*n* = 4)	Overall (*n* = 10)	*p*-value
PT (N)	23.90 ± 7.10*	12.62 ± 2.73	19.39 ± 8.02	*t*_8_ = 2.98, *p* = 0.017
TPT (ms)	51.72 ± 16.03	43.52 ± 16.47	48.44 ± 15.85	*t*_8_ = 0.79, *p* = 0.46
HRT (ms)	51.97 ± 28.28	75.47 ± 32.69	61.37 ± 30.78	*t*_8_ = 1.21, *p* = 0.26
CD (ms)	103.69 ± 41.51	118.98 ± 40.00	109.81 ± 39.41	*t*_8_ = 0.58, *p* = 0.58
PAP (%)	33.20 ± 7.50	27.14 ± 6.39	30.78 ± 7.39	*t*_8_ = 1.32, *p* = 0.22
Voluntary activation (%)	98.5 ± 1.2	97.8 ± 1.6	98.2 ± 1.3	*t*_8_ = 0.85, *p* = 0.42
Elbow Flexor MVC before (N)	196.78 ± 40.43*	107.14 ± 27.44	160.93 ± 57.46	*t*_8_ = 3.85, *p* = 0.005
Elbow Flexor MVC after (N)	185.88 ± 38.75*	103.73 ± 27.78	153.02 ± 53.77	*t*_8_ = 3.63, *p* = 0.007

Values are means ± SD and statistics reported from one-way ANOVA. *, significantly different from females; N, Newton; ms, milliseconds; PT, peak tension; TPT, time to peak tension; HRT, half relaxation time; CD, contraction duration; PAP, post-activation potentiation.

### 3.2 CV of force

**Figure 2:**
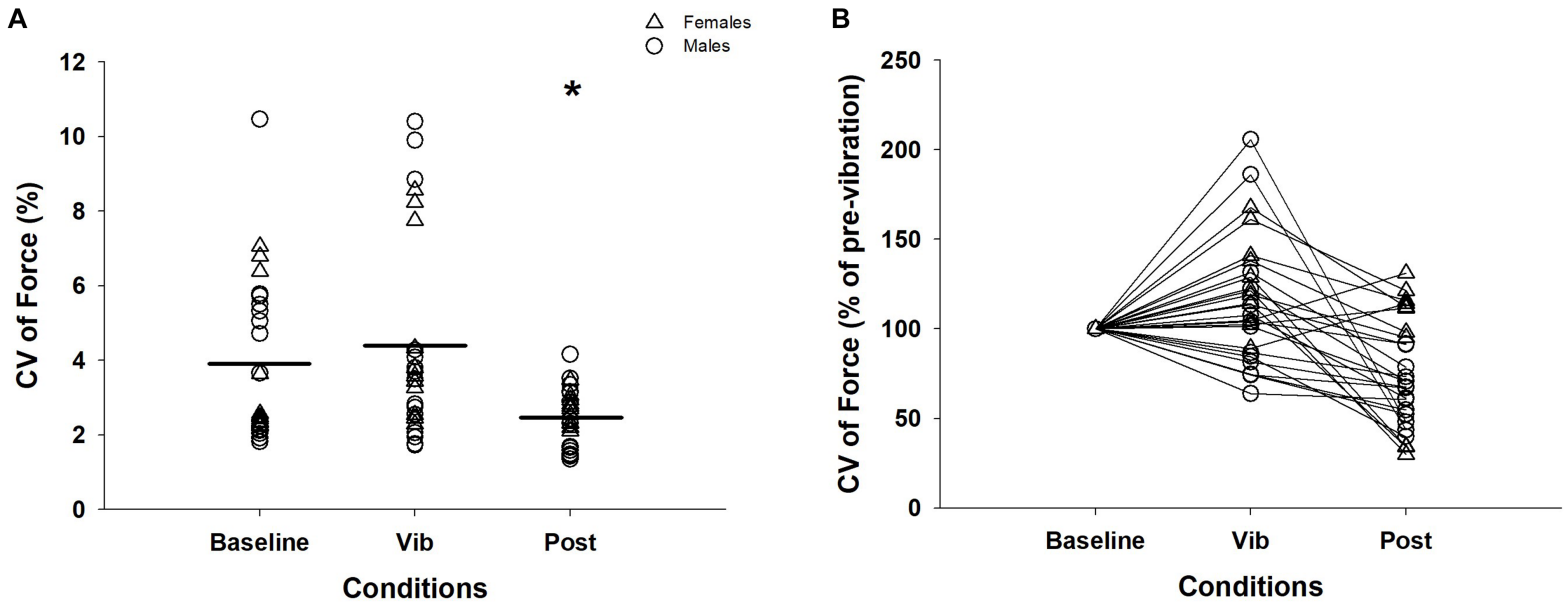
CV of force across three conditions for an isometric contraction at 5%: **(A)** CV of force. There were 3 contractions performed per condition for the 10 participants. **(B)** CV of force normalized to baseline condition for visual purpose. Δ, female; ◯, male; Horizontal line, average of trials; *, significantly different between baseline and vibration (*p* < 0.05); CV, coefficient of variation; Vib, vibration; post, post-vibration.

A Friedman test confirmed a significant effect of vibration on CV of force (χ^2^ (2) = 15.46, *p* < 0.001). Wilcoxon Signed-Rank test results revealed that CV of force during both baseline (3.94 ± 2.19%) and vibration (vib; 4.41 ± 2.61%) conditions were significantly greater than the post-vibration (post: 2.48 ± 0.74%) condition (baseline vs. post: *Z* = −3.75, *p* < 0.001; vib vs. post: *Z* = −4.12, *p* < 0.001) and baseline and vibration conditions were not different (*Z* = –1.37, *p* = 0.17; Figures 2A,B).

### 3.3 Motor unit activity

Recruitment thresholds and derecruitment thresholds of 33 MUs were compared. Derecruitment thresholds were significantly lower than recruitment thresholds (*Z* = −5.00, *p* < 0.001, recruitment: 1.85 ± 1.82%MVC, derecruitment: 0.015 ± 0.016%MVC), and correlation analysis revealed that the MU derecruitment thresholds were positively correlated with the recruitment thresholds (*r* = 0.28, *p* = 0.08). The derecruitment thresholds of all MUs were below 0.05% of MVC regardless of the condition, showing that the MUs were resistant to turning off. The recruitment thresholds and derecruitment thresholds were compared between the three conditions (Table 3), and the average recruitment (baseline:1.59 ± 1.54%MVC, vib:1.77 ± 1.76, post:2.19 ± 2.17; χ^2^(2) = 0.02, *p* = 0.99) and derecruitment thresholds (baseline:0.02 ± 0.02%MVC, vib:0.01 ± 0.01, post:0.02 ± 0.02, χ^2^(2) = 0.40, *p* = 0.82) did not differ between conditions. Yet, the number of MUs which remained active at rest following the contraction were 13 MUs after the baseline condition, 17 following the vibration condition, and 10 following the post-vibration condition (Table 3).

**Table 3:** Average recruitment and derecruitment thresholds.

	Recruitment thresholds (% MVC)	Derecruitment thresholds (% MVC)	MUs recruited and tracked (#)	MUs that did not derecruit (#)	Ratio of recruited to not derecruited (%)
Baseline	1.59 ± 1.54	0.02 ± 0.02	33	13	39.4
Vibration	1.77 ± 1.76	0.01 ± 0.01	33	17	51.5
Post	2.19 ± 2.17	0.02 ± 0.02	33	10	30.3

Identified are the MUs that remained active after the deramp for the three conditions, as well as the ratio of the 33 tracked MUs to those that did not derecruit (sustained firing). Values are means ± SD. #, significantly different from derecruitment thresholds; MUs, motor units; #, number active; % active.

**Figure 3:**
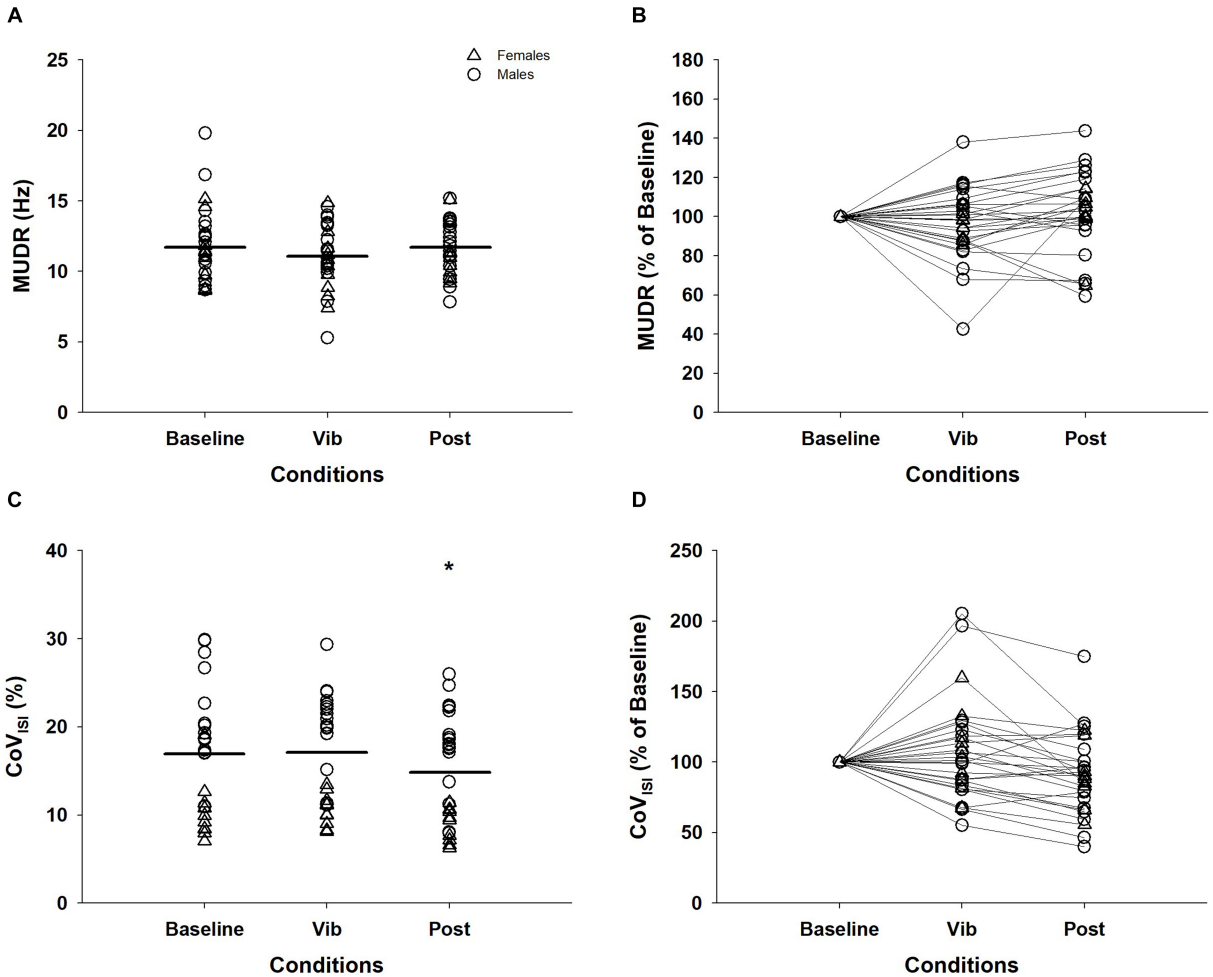
Motor unit discharge rates (MUDR) and motor unit discharge rate variability (CoV_ISI_) of 28 MUs tracked across the plateau of the three conditions. The MUDR **(A)** and CoV_ISI_ **(C)** were normalized to the value of the baseline condition for MUDR **(B)** and CoV_ISI_ **(D)** for visual purposes. Δ, female; ◯, male; Horizontal line, average of individual trials; *, significantly different from baseline and vibration (*p* < 0.05); MUDR, motor unit discharge rate; CoV_ISI_, motor unit discharge rate variabilit; Vib, vibration; post, post-vibration; CoV, coefficient of variation; ISI, interspike interval.

Of the 33 MUs that were consistently detected for recruitment and derecruitment, 28 of these were able to be continuously tracked during the plateau for the three conditions. MUDR (baseline = 11.76 ± 2.63 Hz; vib = 11.13 ± 2.26 Hz; post = 11.61 ± 1.91 Hz) did not differ between the three conditions (χ^2^(2) = 4.57, *p* = 0.10, Figures 3A,B). For CoV_ISI_, a significant effect of vibration was evident (χ^2^(2) = 9.21, *p* = 0.01). CoV_ISI_ during baseline (17.72 ± 6.53%) and vibration (17.87 ± 6.19%) were significantly greater than the post-vibration (14.74 ± 5.91%) condition (baseline vs. post: *Z* = −2.15, *p* = 0.03; vib vs. post: *Z* = −3.38, *p* = 0.001), while the baseline and vibration condition did not differ (*Z* = −0.13, *p* = 0.90; Figures 3C,D).

## 4 Discussion

The objective of this study was to evaluate the effects of acute biceps brachii tendon vibration on force steadiness and MU activity in the elbow flexors of the contralateral and more affected arm of persons with PD. Our primary finding was that force steadiness improved and CoV_ISI_ decreased in the submaximal contractions that followed the application of contralateral tendon vibration. A secondary observation was the number of MUs that were derecruited following post-vibration were greater than in the baseline and vibration conditions.

### 4.1 Contralateral vibration effect

Acute contralateral vibration induced an improvement in force steadiness and decreased CoV_ISI_. This novel finding provides evidence that contralateral vibration might be a non-pharmacological therapeutic tool that can enhance motor function in persons with PD. Specifically, the improvements in force steadiness and decrease in CoV_ISI_ were 37 and 17%, respectively following tendon vibration. Functional disability and impairments in force steadiness at low forces in persons with PD are well established and evident early in disease progression (Corcos et al., 1996; Kandaswamy et al., 2018; Skinner et al., 2019; Smart et al., 2020). The CV of force for persons with PD in this study in the baseline condition was 3.94%, which is two-fold higher than the ~1.5% previously observed in similarly aged healthy older adults in the same experimental setup (Smart et al., 2018). When differences in force steadiness are observed between groups or conditions, absolute strength generally differs (Enoka et al., 2003; Skinner et al., 2019; Smart et al., 2020). The application of vibration culminated in ~1.5x reduction in the CV of force on the contralateral side without a change in MVC suggesting that additional to the role of absolute strength there is a neural locus of influence, rather than an overt and acute increase in force influencing force steadiness (Jakobi et al., 2018). The clinical importance of absolute strength to reduced mobility and quality of life is well established, and cannot be ignored. Absolute strength is a critical aspect of producing steady contractions, and these two features combine to explaining functional ability (e.g., hand-dexterity, balance, gait). The independent contribution of each feature, as well as the combination of features have been evaluated in various regression models, and the coefficient of variation of force during steady contractions seems to explain more of the variance in motor performance than measures of muscle strength (Enoka and Farina, 2021). Yet, together more than 50% of the variance in an array of functional ability measures can be explained by these combined factors in persons with neurological disorders as well as community dwelling older adults (reviewed in 40). Disentangling the independent contribution of each feature needs further research; however, it cannot be dismissed that maximal strength is of paramount importance to remaining functionally independent. In this study contralateral vibration enhanced force steadiness independent of strength and acutely. This offers the potential for a mechanical stimulus to be used in studying the phenomenon of force control. The application and clinical relevance of vibration cannot be assumed, yet observations necessitate future experimental consideration on the duration of effect as well as clinical importance.

We tracked 33 individual MUs between the three conditions, and observed a decrease in MU discharge rate variability without a concomitant change in discharge rate. In healthy adults’ the influence of acute ipsilateral vibration on MU discharge rate variability is equivocal, and this might arise from the contraction intensity and the type of motor units contributing (or recorded) (Germer et al., 2019; Enoka and Farina, 2021). A limitation of this study is the evaluation of one low threshold force, and the lack of a healthy control group. A higher force level would enable assessment of a broader range of MU types and contraction intensities. A healthy control group would enable determination of motor-impairment influence or ‘typical’ spinal integration. Although speculative, the contralateral vibration in this study was unlikely to alter force output and corticospinal drive as is evident with ipsilateral vibration (Grande and Cafarelli, 2003; Lapole et al., 2015b). There is no doubt that the pattern of discharge rate in persons with PD is disrupted (Freund et al., 1973; Dietz et al., 1974; Dengler et al., 1986; Glendinning and Enoka, 1994; Wilson et al., 2020). Independent and common synaptic inputs trigger variability in MU discharge times. Herein, the observation of the decrease in interspike interval variability offers evidence that contralateral vibration induces adaptations in independent inputs (Enoka and Farina, 2021) potentially arising from the commissural system (Maxwell and Soteropoulos, 2020; Kinany et al., 2023). Interestingly, in healthy controls a reduction in variability of the smoothed cumulative spike train was reported (Germer et al., 2020; Lapole et al., 2023) establishing a role for common synaptic input with ipsilateral vibration in diseased and non-diseased states. The mediating effect of ipsilateral and contralateral vibration on discharge rate variability requires specific study design, but all indicators are that vibration is a powerful stimulus to modulate motor units bilaterally.

### 4.2 Sustained MU discharge after contraction completion

In persons with PD sustained MU discharge following a contraction is positively correlated with disease duration, bradykinesia, and rigidity (Grasso et al., 1996). As expected the recruitment threshold (5.85% MVC) of the 33 MUs was higher than the derecruitment (0.05% MVC) threshold. Although the experimental protocol was not designed to systematically quantify MUs after completing a contraction we did observe sustained firing of MUs at rest (following the contraction) in all three conditions. *Post hoc* examination of this observation revealed that the number of MUs that were derecurited were greater following the post-vibration condition. This response, coupled with the decrease in MU discharge rate variability might be indicative of inhibition. Arguably, this could also reflect a delayed adaptation of motor units to the vibration stimulus. We speculate that afferent large fiber sensory input ipsilateral to the vibration stimulus may have effects on diverse circuits including through interhemispheric interactions with the motor cortex, as is known to occur with direct stimulation of sensory areas (Koch et al., 2009). This is seemingly reasonable as contralateral vibration is purported to activate functional connectivity of the ipsilateral and contralateral sensorimotor cortex (Koch et al., 2009; Lapole and Tindel, 2015; Li et al., 2019). The observation of reductions in sustained firing requires systematic study and experimental validation beyond an immediate evaluation following the contractions to establish the etiology of motor unit adaptation to contralateral vibration. What is clear from the literature on ipsilateral vibration and this study on contralateral vibration in persons with PD is that mechanical vibration mediates spinal motoneuron activity and might be useful as a non-pharmacological intervention. Future studies need to explore the duration of influence that vibration offers, and evaluate the potential for tendon vibration to supplement exercise or therapeutic modalities for persons with PD.

### 4.3 Contractile properties

In this study absolute strength was lower than typically observed in non-Parkinsonian persons in the same experimental setup (Smart et al., 2018), yet the reduced MVC was not due to a decrease in voluntary activation. Contractile properties (TPT, HRT, CD) measured in this study are suggestive of typical age-related slowing (Dalton et al., 2010). It is atypical and potentially unsuitable to statistically compare males and females in a small sample. However, biological sex differences are found between females and males in fiber composition and in the dopaminergic pathway, and although disease onset is later in females disability is greater compared with males (Li et al., 2007; Gillies et al., 2014; Abraham et al., 2019). Characterizing and documenting contractile properties in females and males is necessary and appropriate (Schiebinger et al., 2016) and further work is required to understand the sex-specific differences in persons with PD (Roland et al., 2011). More studies in persons with PD are needed to fully appreciate sex-related disease progression and the neurophysiological underpinnings; however, this small sample suggests that in the upper limb decreases in strength are not attributed to contractile failure or declines in voluntary activation in females and males with PD.

## 5 Conclusion

Our results demonstrate that contralateral activation could be an important neurophysiological pathway to improving force steadiness in persons with PD. The decrease in MU discharge rate variability and reduced coefficient of variation of force indicates that force control is enhanced when MU discharge variability is less following a vibration stimulus. More research is needed to isolate the physiological cause and functional implication of using contralateral vibration to improve force steadiness in persons with PD.

## Data availability statement

The raw data supporting the conclusions of this article will be made available by the authors, without undue reservation.

## Ethics statement

The studies involving humans were approved by Clinical Research Ethics Board, The University Research Ethics Board (H11-01931). The studies were conducted in accordance with the local legislation and institutional requirements. The participants provided their written informed consent to participate in this study.

## Author contributions

CK: Writing – review & editing, Data curation, Formal analysis. DW: Writing – review & editing, Conceptualization, Methodology, Resources. SK: Writing – review & editing, Statistical evaluation, Analysis, Appendix writing. KL: Conceptualization, Methodology, Data curation, Formal analysis, Investigation, Project administration, Writing – original draft. JJ: Conceptualization, Investigation, Methodology, Project administration, Writing – original draft, Funding acquisition, Supervision, Writing – review & editing.
