# Supplementary figures and images for: Short term effects of contralateral tendon vibration on motor unit discharge rate variability and force steadiness in people with Parkinson’s disease
Source: Front Aging Neurosci. 2024 Mar 4;16:1301012. doi: 10.3389/fnagi.2024.1301012 (PMC10962053; doi:10.3389/fnagi.2024.1301012)

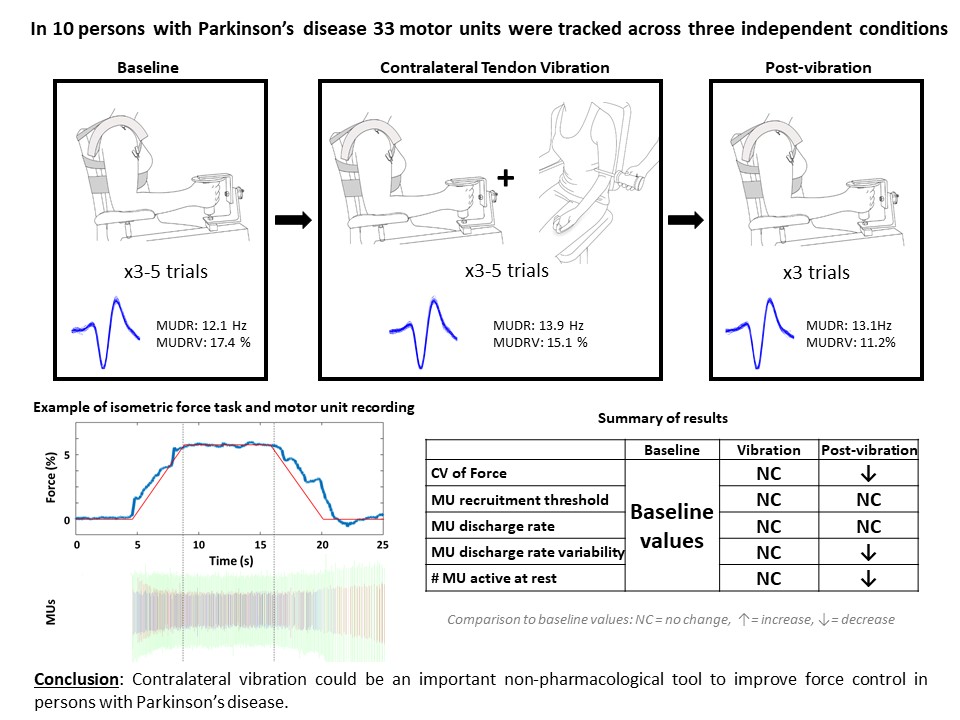

Supplement: Supplementary file 1 [file Image_1.JPEG]
