# Supplementary material for: Short term effects of contralateral tendon vibration on motor unit discharge rate variability and force steadiness in people with Parkinson’s disease
Source: Front Aging Neurosci. 2024 Mar 4;16:1301012. doi: 10.3389/fnagi.2024.1301012 (PMC10962053; doi:10.3389/fnagi.2024.1301012)
Supplement: Supplementary file 2 [file Data_Sheet_1.PDF]

Supplementary Materials

To test if CV of force, MUDR, and MUDRV varied across condition, three separate linear mixed effects (LME) models were conducted using the lme4 package (https://CRAN.R-project.org/package=lme4; Bates et al., 2015). This approach allows data from all motor units as separate observations for each participant and allow testing (and hence control) of both fixed and random (subject) effects (Galwey, 2006). Condition (baseline, entered as the reference level; vibration; post-vibration) was entered as a fixed effect, with participant entered as a random intercept. Effects of condition were additionally interrogated with posthoc contrasts (FDR corrected for multiple comparisons) using the emmeans package (https://CRAN.R-project.org/package=emmeans) comparing each outcome measure (CV of force, MUDR, and MUDRV) between each level of condition.

LME outputs for all analyses are presented in Supplementary Table 1, and results from posthoc analyses are presented in Supplementary Table 2. From results of the LME models and posthocs, we observe differences between post-vibration and baseline, and post-vibration and vibration for both CV of force and MUDRV. No differences were observed related to MUDR.

Supplementary Table 1. *Results from linear mixed effects models testing relationships between CV of force, MUDR, and MUDRV as a function of condition (baseline, vibration, post-vibration). Bold values indicate statistical significance (p < .05).*

	CV of force			***MUDR***			***MUDRV***		
Predictors	*Estimates*	*CI*	*p*	*Estimates*	*CI*	*p*	*Estimates*	*CI*	*p*
(Intercept)	4.31	3.07 – 5.54	**<.001**	11.99	10.82 – 13.16	**<.001**	17.16	13.58 – 20.75	**<.001**
condition [post-vibration]	-1.66	-2.29 – -1.03	**<.001**	-0.01	-0.94 – 0.92	.983	-2.32	-3.98 – -0.66	**.007**
condition [vibration]	0.32	-0.26 – 0.90	.280	-0.42	-1.30 – 0.47	.354	0.19	-1.40 – 1.78	.816
Random Effects									
σ^2	1.41			3.33			10.68		
$\tau_{00\ participant}$	3.43			2.45			29.27		
ICC	0.71			0.42			0.73		
$N_{participant}$	10			10			10		
Observations	94			97			97		
Marginal R^2 / Conditional R^2	0.126 / 0.745			0.007 / 0.428			0.031 / 0.741		

Supplementary Table 2. *Post hoc contrasts comparing CV of force, MUDR, and MUDRV for effects of condition using the emmeans package in R programming environment. Bold values indicate statistical significance (p < .05, FDR corrected for multiple comparisons).*

	CV of force			*MUDR*			*MUDRV*		
Contrast	*Estimate*	*SE*	*p-FDR*	*Estimate*	*SE*	*p-FDR*	*Estimate*	*SE*	*p-FDR*
baseline – post-vibration	1.659	0.317	**<.0001**	0.0101	0.467	.9828	2.319	0.836	**.0102**
baseline -vibration	-0.316	0.291	.2807	0.4160	0.447	.5812	-0.187	0.801	.8163
post-vibration - vibration	-1.975	0.318	**<.0001**	0.4060	0.467	.5812	-2.506	0.837	**.0102**

References

Bates D, Mächler M, Bolker B, Walker S (2015). "Fitting Linear Mixed-Effects Models Using lme4." *Journal of Statistical Software*, **67**(1), 1–48. doi:10.18637/jss.v067.i01.

Galwey, N. W. (2006). Introduction to Mixed Modelling: Beyond Regression and Analysis of Variance. Hoboken, NJ: Wiley
